# Comparative Analysis of Selected Chemical Parameters of *Coffea arabica*, from Cascara to Silverskin

**DOI:** 10.3390/foods11081082

**Published:** 2022-04-08

**Authors:** Alica Bobková, Katarína Poláková, Alžbeta Demianová, Ľubomír Belej, Marek Bobko, Lukáš Jurčaga, Branislav Gálik, Ivana Novotná, Amaia Iriondo-DeHond, María Dolores del Castillo

**Affiliations:** 1 Institute of Food Sciences, The Faculty of Biotechnology and Foods Sciences, The Slovak University of Agriculture in Nitra, Tr. A. Hlinku 2, 94976 Nitra, Slovakia; alica.bobkova@uniag.sk (A.B.); xdemianova@uniag.sk (A.D.); lubomir.belej@uniag.sk (L’.B.); marek.bobko@uniag.sk (M.B.); xjurcaga@uniag.sk (L.J.); 2 Institute of Nutrition and Genomics, The Faculty of Biotechnology and Foods Sciences, The Slovak University of Agriculture in Nitra, Tr. A. Hlinku 2, 94976 Nitra, Slovakia; branislav.galik@uniag.sk (B.G.); ivana.novotna@uniag.sk (I.N.); 3 Instituto de Investigación en Ciencias de la Alimentación (CIAL) (CSIC-UAM), Campus de la Universidad Autónoma de Madrid, C/Nicolás Cabrera, 9, 28049 Madrid, Spain; amaia.iriondo@csic.es (A.I.-D.); mdolores.delcastillo@csic.es (M.D.d.C.)

**Keywords:** coffee, Cascara, dietary fiber, dry matter, total fat, fatty acids, coffee beans, roasting process, coffee Silverskin

## Abstract

Nowadays, there is an increased interest in coffee derivatives (green beans, roasted beans, and coffee by-products (Cascara and Silverskin)) due to their particular chemical composition. This study aimed to compare the content of dry matter, total fat, fatty acids, and fiber (ADF, NDF) of coffee by-products (Cascara and Silverskin) and coffee beans (green and roasted under different conditions). Coffee beans and their by-products were obtained from 100% *C. arabica* coffee cherries from Panama by dry process. The lowest concentrations of fat corresponded to Cascara 4.24 g·kg^−1^ and Silverskin 23.70 g·kg^−1^, respectively. The major fatty acids detected in all samples were palmitic, stearic, oleic, and linoleic acids, the latter two being essential fatty acids. LDA showed that 89.01% of the variability between beans and by-products was explained by lignoceric, myristic, behenic, tricosanoic, arachidic, and heneicosanoic acids. Silverskin appeared to be a good source of lignoceric, myristic, and behenic acids and had a higher concentration of dietary fiber (314.95 g·kg^−1^) than Cascara (160.03 g·kg^−1^). Coffee by-products (Silverskin and Cascara) are low-fat products enriched in dietary fiber. Their incorporation, after adjustment, into the global diet may contribute to nutrition security, the sustainability of the coffee sector, and human health.

## 1. Introduction

With constantly increasing consumption, coffee beans are one of the most traded commodities globally [1]. According to Iriondo-DeHond et al. (2019) [2], a coffee cherry has various anatomic parts characterized by unique chemical and bioactive compositions. A coffee cherry consists of the outer skin, usually green in unripe fruit and red (some particular genotypes can be orange or yellow) in ripe fruit, which covers a soft and sweet flesh, following the highly hydrated layer of mucus (pectin layer), a thin yellowish endocarp, parchment, and finally the Silverskin covering the green coffee beans [3,4].

The post-harvest processing aims to obtain green coffee beans. The first step of processing starts with the pulp and hull removal using either a wet or dry method [5]. The dry and wet processing methods affect the composition of coffee beans and their by-products [6]. Harvested coffee cherries are dried in the sun and hulled using the mechanical system during the dry process. The dried husk, consisting of skin, pulp, mucilage, and parchment, is removed, together with as much Silverskin as possible [4]. Wet processing depulper removes the pulp. Then, the fermentation takes place to eliminate mucilage. Next, the beans covered by the parchment are washed, drained, and dried until the moisture reaches around 10%. In the final step, the parchment is removed using a hulling machine. Out of 100 kg of mature coffee cherries, 39 kg of pulp and skin is produced [7].

The chemical composition of the green beans primarily depends on genetic variation, post-harvest processing techniques, bean maturation, agricultural practices, and environmental gradients. The roasting process determines coffee sensory characteristics [8,9,10]. During the roasting, hundreds of physical and chemical reactions occur.

The coffee industry produces by-products rich in bioactive compounds such as polyphenols, alkaloids, chlorogenic acids, antioxidants, carbohydrates, proteins, and dietary fiber. These by-products can be a problem for the environment, roasters, growers, and producers, given that they have no further use [2]. Approximately 90% of the coffee cherry parts are discarded and classified as agricultural waste. Cascara or husk, mucilage, parchment, Silverskin, and spent coffee grounds are the main by-products [3]. The latter is produced during the brewing process, and it is also composed of nutrients present in roasted coffee beans such as fatty acids and insoluble dietary fiber (cellulose and hemicellulose) [11,12,13]. Spent coffee grounds also contain micronutrients (potassium, calcium, magnesium, sulfur, phosphorus, iron, manganese, boron, and copper) [14]. Consequently, they have been exploited as a value-added food product [15].

**Figure 1:**
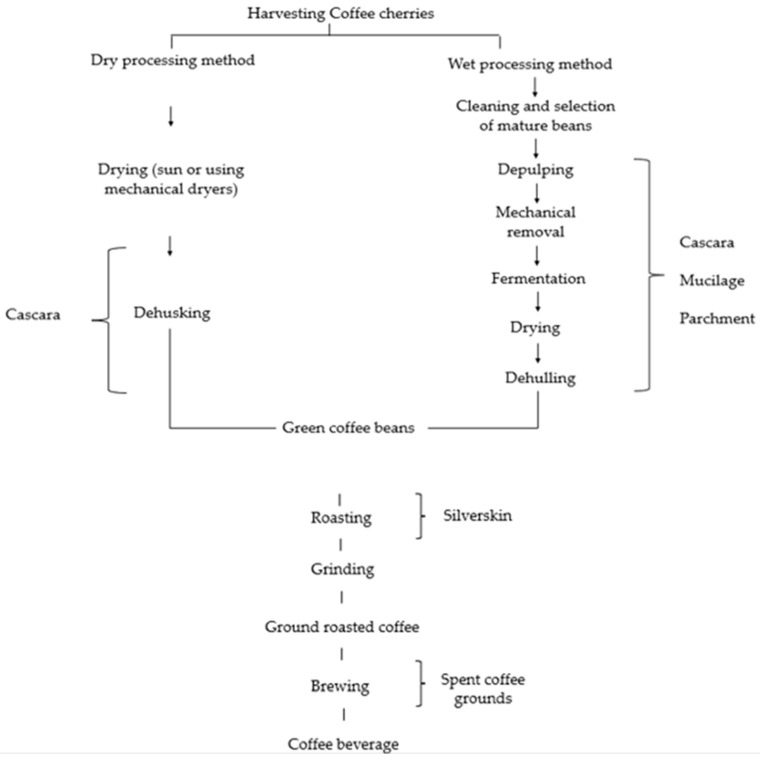
Schematic representation of coffee processing and by-products generation adapted from [16].

Based on Figure 1 [16] it is essential to distinguish between the cherry pulp and cherry husk (Cascara). These products are obtained by using different methods of coffee bean processing [1]. Coffee by-products have been designed as a potential sustainable source of macronutrients, micronutrients, and bioactive compounds. Due to the chemical composition of green coffee beans, a similar composition could be assumed for coffee by-products. Cascara and Silverskin are considered to be good sources of dietary fiber. Cascara has potential as a natural sustainable source of nutrients, which include proteins (8–11%), minerals (3–7%), lipids (0.5–3%), and total carbohydrates (58–85%), cellulose (24.5%), hemicellulose (29.7%), and lignin (23.7%) [1,3,17].

Silverskin is detached from green beans and represents the only by-products produced during the roasting process. One hundred and twenty kilograms of green coffee generate approx. 2.5 kg of Silverskin [6]. Silverskin contains a higher dietary fiber level (cellulose, hemicellulose, and lignin) compared to coffee beans. Xylose, arabinose, galactose, and mannose are the main monosaccharides in Silverskin [18]. Dietary fiber (55%) is the significant component of Silverskin, which includes soluble (10%) and insoluble (45%) fiber. Part of the dietary fiber composing coffee Silverskin is soluble, which gives the potential to develop functional foods [3]. Other significant components are proteins (19%), carbohydrates (6%), and fat (6%). Silverskin is also a rich source of polyphenols, chlorogenic acid, and antioxidants [2,11].

Several studies describe coffee by-products’ health benefits, mainly antioxidant, antimicrobial, anti-inflammatory, and anti-aging effects, while very few data have been provided on the nutritional value of them. Moreover, to the best of our knowledge, there are no previous papers available comparing the nutritional composition of unroasted and roasted coffee beans and the by-products generated during post-harvest processes such as depulping (Cascara) and roasting (Silverskin). Previous studies on the content of food contaminants, (ochratoxin A) and acrylamide [18,19,20], support the food safety of coffee by-products for human consumption. Consequently, the EFSA (2021) [21] has considered Cascara a traditional food that can be commercialized in Europe for human consumption, starting from January 2022. Therefore, the upcycling of coffee by-products allows for the reduction of their environmental impact and turns them into nutritious ingredients and foods that can contribute to nutrition security and sustainable health.

The aim of the present research is to provide novel information on the nutritional composition of coffee beans and the by-products generated during coffee post-harvest processing. We applicated a statistical comparison of the fatty acid profile in green coffee beans, their roasted forms (light, medium, and dark), and coffee by-products (Cascara and Silverskin). A comparative study on their chemical composition was performed with the end to stimulate their inclusion as part of a daily healthy diet for humans. At the same time, the paper aims to contribute to the sustainability of the coffee sector and nutrition security. The intake of the chemicals selected for analysis includes nutrients (fats and dietary fiber) that should be controlled to avoid the risk of non-communicable chronic diseases considered pandemics of the 21st century.

## 2. Materials and Methods

### 2.1. Material

For this research, Cascara and green bean samples were obtained from *Coffea arabica* cherries from Central America, Panama, Hartmann micro-lot, Santa Clara, by dry method (natural) of post-harvest processing. Silverskin was obtained as mixture from light, medium, and dark roasting processes. The samples were obtained from the local company dedicated to importing and distributing coffee, Caffé Oro Ltd. (Zvolen, Slovakia). All measurements were performed in triplicate and results are shown in g·kg^−1^ of dry matter.

### 2.2. Roasting Process

Green coffee was roasted using traditional barrel roaster technology with a gas heating system using Toper TKSMX 10 machine (Toper, Izmir, Turkey). Roasting programs were set as follows: Light roast (Cinnamon)—temperature 180 °C for 3 min, then 207 °C for 2 min and 50 s. Medium roast (Full City++)—the initial temperature was 180 °C for 3 min, then after 4 min the first crack occurred at 205 °C and ended at 215 °C. The second crack started at 225–230 °C (Full City +). Five seconds later, the beans reached 232 °C for 15 s. Dark roast (French)—the initial temperature was 180 °C for 3 min, then after 4 min the first crack occurred at 205 °C and ended at 215 °C. The second crack started at 225–230 °C. Then, we kept the beans at 235 °C for 45 s; samples of Silverskin were obtained from the roasting process. Silverskin was collected after each roasting process, and coffee beans were placed in a metal container to cool to room temperature, then moved to odor-free plastic bags with a CO_2_ de-gassing valve obtained from Caffé Oro Ltd. Samples were analyzed the next day. To ensure that the right roasting level was reached, roasted samples were measured using RoastRite, Coffee Roast Analyzer (RoastRite^TM^, Taipei City, Taiwan). A detailed description of samples is shown in Table 1.

### 2.3. Grinding

Given the hard structure of green beans, samples were frozen at −20 °C for 30 min before the analysis to ensure easier homogenization. Samples were homogenized using Grindomix GM 200 (Retsch, Haan, Germany) for 60 s (roasted beans, Cascara, and Silverskin) and 120 s (green beans) at 10,000 rpm to achieve the required grinding thickness. The homogenized samples were then sieved through a sieve with a mesh diameter of 1 mm.

### 2.4. Determination of Dry Matter

The dry matter was determined by moisture analyzer KERN DAB 100-3 (KERN & SOHN GmbH, Balingen, Germany) and expressed in g·kg^−1^.

### 2.5. Fat and Fatty Acids Profile

Total fat content was quantified by Soxhlet extraction with petroleum ether following the procedure described by the ISO 12966-2:2017: preparation of methyl esters of fatty acids, animal and vegetable fats, and oils [22]. Gas chromatography of fatty acid methyl esters was employed for the analysis of the individual profile. The fat content and fatty acids methyl ester profile are in g·kg^−1^ of dry matter.

- Chemical reagents

Petroleum ether 40–65 °C (Sigma-Aldrich, Steinheim, Germany), filter medium Hyflo Super Cel (diatomaceous earth) (Sigma-Aldrich, Steinheim, Germany), methanol for chromatography (Sigma-Aldrich, Steinheim, Germany), KOH (Centralchem, Bratislava, Slovakia), HCl 33–36% (Centralchem, Bratislava, Slovakia), NaHSO_4_ H_2_O (Centralchem, Bratislava, Slovakia), Na_2_SO_4_ (Centralchem, Bratislava, Slovakia), anhydrous N-hexane for chromatography (Sigma-Aldrich, Steinheim Germany), 37 component standard FAME mix 10 mg.mL^−1^ in methylene chloride containing C4-C24 FAME (2–4% relative concentration), manufacturer Supelco, catalog number 47885-U chromatography (Sigma-Aldrich, Steinheim, Germany).

- Apparatus

1. Tecator Soxtec System HT 1043 Extraction unit (Gemini, Apeldoom, the Netherlands) apparatus at a flow rate of 10 cycles/h or 10 mL·min^−1^;

2. Vacuum oven, (Fisher Scientific, Waltham, MA, USA) temperature adjustable (100 ± 3 °C).

#### Preparation of Fatty Acid Methyl Esters (FAME)

The fat used must be liquid, dry, and clean. Approximately 200 mg of fat was weighed into a ground glass tube. A total of 5 mL of n-hexane was added to the fat by pipette. The fat was dissolved by stirring. In total, 1 mL of 2 M KOH in methanol was added with a micropipette. The tube contents were shaken vigorously and placed in a water bath set at 60 °C for 1 min and 30 s. Then, 2 mL of 1 M HCl was added, and the tube contents were gently mixed. After equilibration and separation of the layers, the upper layer containing FAME was carefully removed by pipette, filtered through anhydrous Na_2_SO_4_, and used for GC-FID analysis. Prior to loading into the column, the FAME solution was diluted in a vial, usually at a ratio of 50 μL FAME solution/950 μL n-hexane.

GC-FID was performed using Agilent 6890 GC with FID (flame ionization detector), and analytical column: 60 m × 250 μm × 0.15 μm DB-23 (Agilent 122-2361) was used. Experimental conditions were set as follows: injector temperature: 250 °C; injected volume: 1 μL; dividing ratio: 1/10; carrier gas: helium; head pressure: 238.96 kPa (2.225 mL·min^−1^); temperature program: 50 °C for 1 min, 25 °C min^−1^ till the system reached 175 °C, then 2 °C min^−1^ to 230 °C; detector temperature: 280 °C; gas detector: hydrogen: 35 mL·min^−1^, air: 350 mL·min^−1^, nitrogen; make-up gas: 30 mL·min^−1^.

The quality indicator was the elution time of the separated analytes. The chromatograms of the samples were compared with the chromatogram of the standard. The quantity indicator is the area under the peak of the analyte of interest.

The internal normalization method was used for quantitative evaluation, i.e., provided that all components of the sample were recorded on the chromatogram, representing a total peak area of 100%, and the areas under the individual peaks, i.e., the individual fatty acid methyl esters, represented the weight percentage of a given fatty acid of the total fatty acid content present in the sample [23].

### 2.6. Determination of Dietary Fiber, Neutral Detergent Fiber (NDF), and Acid Detergent Fiber (ADF)

The analysis was performed according to the Decree of the Ministry of Justice of the Slovak Republic no. 2145/2004-100 of 23.8.2004 on official sampling and laboratory testing and evaluation of feeding stuff [23]. The procedure is described in detail in the Supplementary Material.

### 2.7. Statistical Analysis

For the summarizing and describing of our results, descriptive statistics was performed. The ANOVA (Duncan test and REGWQ) and linear discriminant analysis (LDA) were used to describe any possible significant differences between the analyzed samples. This statistical analysis was performed using Microsoft Office Excel 365 for iOS and XLStat, Addinsoft (2022). XLStat is a statistical and data analysis solution (New York, NY, USA).

LDA is a statistical method based on reduction of a classification. This common technique provides class separability by drawing a decision region between the different classes. This advanced method focuses on pattern recognition and machine learning to find a linear combination of characteristics that separates observations into classes, which is used for dimensionality reduction and better classification (maximizing the distance between the means of two classes, minimizing the variation between each category) [24,25].

## 3. Results and Discussion

### 3.1. Dry Matter (DM)

Green beans reached an average DM of 932.20 g·kg^−1^. Our results indicated a relationship between roasting degree and DM values. Under dark roasting conditions, the highest values of DM were obtained, being on average 983.10 g·kg^−1^. Medium and light values were 981.20 and 959.85 g·kg^−1^, respectively. Our previous research showed that medium roasted coffee could have from 967.7 to 998.2 g·kg^−1^ of DM [26]. Results agreed with those reported by Król et al. (2019) [27], which were of 962.4 ± 8.7 g·kg^−1^ for light, 982.8 ± 10.4 g·kg^−1^ for medium, and 987.4 ± 9.1 g·kg^−1^ dark roasting conditions. Cascara DM value was of 890.5 g·kg^−1^, similar to 911.3 ± 9.66 g·kg^−1^ reported by Ameca et al. (2018) (911.3 ± 9.66 g·kg^−1^) [28]. The Silverskin mix showed an average DM value of 920.80 g·kg^−1^. Significant differences were observed between each analyzed group (*p* < 0.0001).

### 3.2. Fat and Fatty Acids Methyl Esters (FAME)

The fat content of the samples in decreasing order was as follows: DRC (135.84 g·kg^−1^) > GC (99.42 g·kg^−1^) > MRC (73.22 g·kg^−1^) > LRC (68.64 g·kg^−1^) > SS (23.70 g·kg^−1^) > CC (4.24 g·kg^−1^). The results indicated that fat content increased in relation to the roasting process. Endeshaw and Belay (2020) [29] showed a similar trend, with the fat content in dark roasted coffee beans being higher than those found in medium or light roasted coffees. Liu and Kitts (2011) [30] associated this behavior to the degradation of carbohydrates and the evaporation of volatile chemicals.

Regarding coffee by-products, our results are in accordance with the findings presented by Iriondo-DeHond et al. (2020) [3], who reported low levels of fat in Cascara and Silverskin samples. Costa et al. (2018) [31] described that Silverskin contains 24.2 g·kg^−1^ of fat. Similar results were obtained in our research. Results and significant differences based on ANOVA Duncan and REGWQ tests are shown in Table 2. Chromatograms of GC-FID analysis of the coffee beans and their by-products are shown in Supplementary Material Figure S1.

Thirteen acids of the 40 analyzed were detected in the coffee and/or its by-products (Table 2). The rest—arachidonic acid, butyric acid, capric acid, caproic acid, caprylic acid, cis-10-heptadecenoic acid, cis-10-pentadecenoic acid, cis-11,14,17-eicosatrienoic acid, cis-11,14-eicosadienoic acid, cis-13,16-docosadienoic acid, cis-5,8,11,14,17-eicosapentaenoic acid (EPA), cis-8,11,14-eicosatrienoic acid, elaidic acid, erucic acid, lauric acid, linolelaidic acid, myristoleic acid, nervonic acid, cis-4,7,10,13,16,19-docosahexaenoic acid (DHA), palmitoleic acid, pentadecanoic acid, tridecanoic acid, undecanoic acid, and γ-linolenic acid—were not identified in the coffee beans nor in the by-products.

Linoleic acid was the most abundant fatty acid in the samples under study, which in decreasing order are as follows: (60.56 g·kg^−1^) > GC (43.67 g·kg^−1^) > MRC (32.54 g·kg^−1^) > LRC (30.19 g·kg^−1^) > SS (5.88 g·kg^−1^) > CC (1.32 g·kg^−1^) and palmitic acid DRC (47.61 g·kg^−1^) > GC (34.93 g·kg^−1^) > MRC (25.39 g·kg^−1^) > LRC (24.40 g·kg^−1^) > SS (6.03 g·kg^−1^) > CC (1.34 g·kg^−1^). Dong et al. (2015) [32] stated that linoleic, palmitic, oleic, and arachidic acid are typical for coffee. Other acids identified in coffee are myristic, stearic, and linolenic [33]. Our results are in accordance with those previously reported [34], which indicated as major components palmitic and linoleic acids in Silverskin. The same study identified low values of heptadecanoic, lignoceric, and myristic acids in this by-product.

We found similar concentrations of oleic and stearic acids in green coffee beans: 7.23 g·kg^−1^ of oleic and 6.96 g·kg^−1^ of stearic acid, respectively. After light and medium roasting processes, we observed a significant decrease of both fatty acids. However, under dark roasting conditions their concentration significantly increased compared to the green beans. Coffee by-products contained significantly lower values (Cascara 0.24 g·kg^−1^ and Silverskin 1.12 g·kg^−1^) than those found in green and roasted coffee beans. Tsegay et al., 2020 [35], found a content of 55.5 g·kg^−1^ of palmitic acid, 8.92 g·kg^−1^ of oleic acid, and 51.6 g·kg^−1^ of linoleic acid in green coffee beans. They reported a relationship between the concentration of fatty acids and geographical origin, namely the altitude of the growing area. Our results are in accordance with this research (Table 2). The roasting process caused a significant decrease in arachidic acid. Similarly, Koshima et al., 2020 [33], observed the same effect. Interestingly, Silverskin contained 13.70% significantly higher concentrations of this acid compared with that found in the green beans.

**Figure 2:**
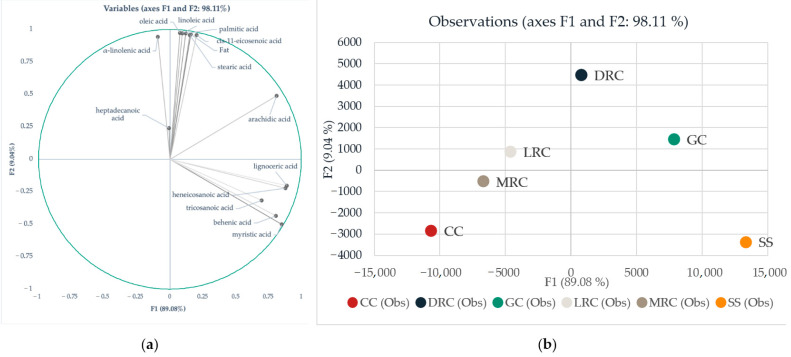
Differentiation of coffee and its by-products based on FAME using linear discriminant analysis (LDA): (**a**) variables representation and mutual correlation in 2-dimensional LDA and (**b**) LDA map where: GC = green coffee, LRC = light roast coffee, MRC = medium roast coffee, DRC = dark roast coffee, CC = Cascara, SS = Silverskin.

The chemical composition of green, roasted coffee and its by-products is crucial from a nutrition point of view and for further describing potential differences between coffee and its by-products to determine the possible application in producing novel, innovative foods. To better understand mutual correlations, variability, and pattern recognition, we subjected data to linear discriminant analysis. This type of statistics was previously used for similar purposes, mainly for pattern recognition and differences description regarding geographical origin [36,37]. Wilks’s lambda test showed that at least one mean vector is different with strong significance (*p* < 0.0001). According to our results, 98.11%, thus the majority, of variability can be described by two factors (Figure 2a). Vectors showed in Figure 2a are responsible for the location of each analyzed group in the 2D space. The higher the content of individual acids is, the stronger their corresponding vectors are in sLDA.

Factor F1 explains 89.08% of the variability and correlates with lignoceric, myristic, behenic, tricosanoic, arachidic, and heneicosanoic acids. Furthermore, Factor F2 explains almost the rest of the variability, 9.04%, and correlates with fat content, heptadecanoic, α-linolenic, oleic, linoleic, palmitic, cis-11-eicosenoic, and stearic acids (Figure 2a).

According to LDA (Figure 2), green coffee is a good source of arachidic acid, compared to roasted coffee. On the other hand, light and medium roast processing caused a significant decrease in each identified fatty acid. Only behenic, cis-11-eicosenoic, and lignoceric acids showed stability in their content under light and medium roasting conditions. Variable representation showed that dark roasted beans are a better source of fat, stearic, palmitic, linoleic, oleic, cis-11-eicosenoic, and α-linolenic acids, compared to the green, light, and medium roasted beans. On the contrary, our results suggest that coffee Cascara is not a rich source of the above-mentioned acids. Cascara was defined mostly by linoleic and palmitic acids in smaller concentrations than those found in green and roasted beans. However, Silverskin appeared to be a good source of lignoceric, myristic, and behenic acids.

The identified fatty acids were divided into three groups (PUFA, MUFA, and SFA). Polyunsaturated fatty acids (PUFA) are defined as hydrocarbon chains containing two or more double bonds. The characterization either as an n-3 PUFA or n-6 PUFA refers to the position of the first double bond relative to the methyl end of the fatty acid. In nature, double bonds are usually in the cis form [38]. Monounsaturated fatty acids (MUFA) are chemically classified as fatty acids containing a single, double bond [39]. On the other hand, saturated fatty acids (SFA) have no double bond; the human body can synthesize this type of fat [40].

**Figure 3:**
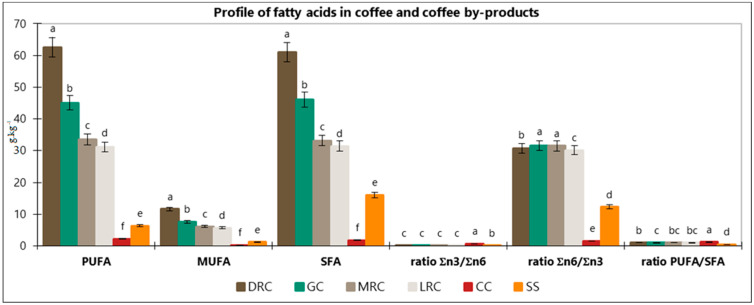
ANOVA differences in PUFA, MUFA, SFA, ratio Σn3/Σn6, ratio Σn6/Σn3, and ratio PUFA/SFA. Different letters denote significant statistical differences (*p* ≤ 0.0001) between samples for a particular group of lipids. GC = green coffee, LRC = light roast coffee, MRC = medium roast coffee, DRC = dark roast coffee, CC = Cascara, SS = Silverskin.

Figure 3 summarizes differences in fatty acid profiles of the samples. As can be observed, PUFA (linoleic and α-linoleic), MUFA (oleic and cis 11-eicosenoic acids), and SFA (myristic, palmitic, heptadecanoic, stearic, arachidic, heneicosanoic, behenic, tricosanoic, and lignoceric acids) contents were significantly higher (*p* < 0.001) in coffee beans compared to their by-products.

PUFA content in green coffee was 45.05 g·kg^−1^. The content of PUFA in roasted beans increased during the roasting process (LRC < MRC < DRC). The lowest concentration of PUFA was observed in coffee by-products (Cascara 2.20 g·kg^−1^; Silverskin 6.35 g·kg^−1^). Omega-3 and omega-6 fatty acids are essential PUFA (polyunsaturated fatty acids). These acids must be a part of our diet, given that they cannot be synthesized in the human body. The balance between dietary n-6 and n-3 fatty acids is crucial regarding human health [41,42]. PUFA, especially linoleic and α-linolenic acids, affect the function and responsiveness of cell membranes and tissue metabolism, to hormonal and other signals. These biological activities may be grouped as the regulation of membrane structure and function, regulation of intracellular signaling pathways, transcription factor activity together with gene expression, and regulation of the production of bioactive lipid mediators. Through these actions, fatty acids affect health, well-being, and the risk of developing disease [41,42,43].

Green coffee beans contained 7.56 g·kg^−1^ of monounsaturated fatty acids (MUFA). The roasting process significantly increased their content. The highest concentration of MUFA reached beans roasted on dark level (11.60 g·kg^−1^). As expected, Silverskin (1.21 g·kg^−1^) and Cascara (0.24 g·kg^−1^) showed low levels of them. Schwingshackl and Hoffmann (2012) [39] stated that data from meta-analyses exploring evidence from long-term perspective cohort studies provide ambiguous results with respect to the effects of MUFA on the risk of coronary heart disease (CHD). However, several studies have indicated an increase of HDL cholesterol and a corresponding decrease in triacylglycerols following a MUFA-rich diet.

The values of SFA copied the following decreasing order: DRC (61.06 g·kg^−1^) > GC (46.09 g·kg^−1^) > MRC (33.16 g·kg^−1^) > LRC (31.46 g·kg^−1^) > SS (16.01 g·kg^−1^) > CC (1.80 g·kg^−1^).

The Σn6/Σn3 ratio in green coffee reached 31.57 g·kg^−1^. The range of the Σn6/Σn3 ratio in the roasted samples ranged from 30.17 g·kg^−1^ (LRC) to 31.48 g·kg^−1^ (MRC). Silverskin contained higher values (12.29 g·kg^−1^) of Σn6/Σn3 ratio compared to the Cascara sample. The values of the n3/n6 ratio in green, light, medium, and dark were identical (0.03 g·kg^−1^). The observed difference of the ratio n3/n6 was in coffee by-products (Silverskin 12.29 g·kg^−1^; Cascara 0.67 g·kg^−1^).

The PUFA/SFA ratio is known to be a crucial parameter regarding human health and nutrition, given that specific saturated and polyunsaturated fatty acids might have different effects in metabolism. Foods with a low PUFA/SFA rate are considered unhealthy and may induce hypercholesterolemia. When this ratio is analyzed separately, there are some restrictions, considering all SFA can increase blood cholesterol, thus ignoring the hypocholesterolemic effect of MUFA [44]. Foods with Σ PUFA/Σ SFA ratios below 0.45 have been considered undesirable for human diet because of their potential to induce a cholesterol increase in the blood [45]. The ratio in green and light roasted samples reached 0.97 and 0.99, respectively. Given that the medium roasting process caused significant changes in fatty acids (Table 2), the PUFA/SFA ratio in medium and dark roasted beans increased from 1.01 (medium) to 1.02 (dark), but the changes were not significant (Figure 3). Among the by-products, Cascara reached the highest ratio of PUFA/SFA, 1.23, while Silverskin showed a significantly lower value of 0.44. Consequently, the lipid profile of the different coffee products studied in the present investigation can be considered healthy.

**Figure 4:**
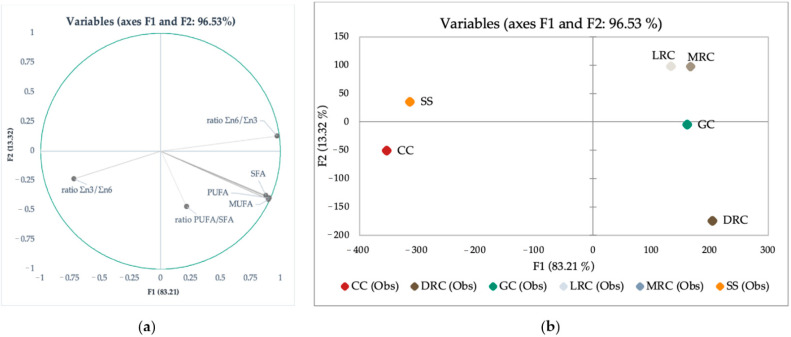
LDA map for PUFA, MUFA, SFA, ratio Σn3/Σn6, ratio Σn6/Σn3, and ratio PUFA/SFA): (**a**) variable’s representation and mutual correlation in 2-dimensional LDA and (**b**) LDA map where: GC = green coffee, LRC = light roast coffee, MRC = medium roast coffee, DRC = dark roast coffee, CC = Cascara, SS = Silverskin.

Figure 4 shows the graphical representation of the LDA of fatty acid profiles and PUFA ratios. In this case, Wilks’s lambda test shows that at least one mean vector is different with strong significance (*p* < 0.0001). Two factors are needed to explain the majority (95.24%) of the variability between green, roasted coffee beans, and their by-products (Silverskin and Cascara). Factor F1 explains 82.28% of the variability and correlates with PUFA, MUFA, SFA, and ratio Σn6/Σn3. Factor F2 is responsible for 12.96% variability and correlates with Σn3/Σn6 (Figure 4a). Based on the LDA map, it was evident that green and roasted coffees are well separated from their by-products, Cascara and Silverskin (Figure 4b). Given the vector attributes, Figure 4a suggests that PUFA, MUFA, and SFA are more characteristics for coffee beans (green beans and dark roast coffee beans).

### 3.3. Determination of Dietary Fiber, ADF, and NDF

Table 3 shows data on the dietary fiber of the samples under study. Green coffee beans from Panama contained 167.29 g·kg^−1^ of dietary fiber. We observed the increase caused by the roasting degree, with the medium level (MRC) having the highest values, 284.59 g·kg^−1^, which is higher by 25% than the dark roasting level (DRC 213.45 g·kg^−1^). Similarly, Endeshaw and Belay (2020) [29] reported that the fiber contents in roasted coffee could range from 13.13 to 33.10%, and the fiber content of dark roasted coffee was lower than in medium and light roasted coffee. The results of the fiber by Ranic et al. (2015) [46] showed that in roasted coffee it is 26.5 ± 2.8 g·100 g^−1^.

Costa et al., 2018 [31], determined that Silverskin has approximately 564 g·kg^−1^ of fiber. Iriondo-DeHond et al. (2020) [3] reported that Cascara and Silverskin are main sustainable sources of dietary fiber. Our results showed that the highest fiber content reached was with Silverskin (SE 314.95 g·kg^−1^); however, Cascara had only 88.34 g·kg^−1^ of fiber.

Holtzapple 2003 [47] stated that acid-detergent fiber is a residue containing cellulose, lignin, and insoluble minerals (mainly silica). Neutral detergent fiber (NDF) is the total cell wall, which is comprised of the ADF fraction plus hemicellulose. Acid detergent fiber (ADF) refers to the cell wall portions that are made up of cellulose and lignin [47,48].

A similar pattern as in the fiber was observed within ADF and NDF, which means that the roasting process caused a significant increase (*p* < 0.0001) in both parameters. Nevertheless, the dark roast caused a significant decrease (*p* < 0.0001). The medium roasting level reached the highest value of ADF and NDF, 41.82 g·kg^−1^ and 60.45 g·kg^−1^, respectively.

The by-products reached relatively high values of ADF and NDF too. When comparing the content of ADF, we observed that Silverskin contained 38.64 g·kg^−1^, whereas Cascara only contained 18.03 g·kg^−1^. The same pattern was observed for NDF. Ameca et al. (2018) [28] evaluated ADF and NDF in the Cascara obtained from *C. arabica* from Mexico. They observed that samples contained 52.14% of ADF and 55.19% of NDF. However, their samples of Cascara were obtained using wet post-harvest processing. Klingel et al. (2020) [18] had similar results when focusing on the fiber in Silverskin and Cascara. Rios et al. (2020) [49] also identified total dietary fiber (474.44 ±18.5 g·kg^−1^), insoluble dietary fiber (313.2 ± 15.1 g·kg^−1^), and soluble dietary fiber (161.2 ± 11.5 g·kg^−1^) in Cascara. Guglielmetii et al. (2019) [50] state that Silverskin extract is a good source of total dietary fiber (36.06 ± 1.67 g.100g^−1^).

## 4. Conclusions

The roasting degree differently affected the composition of the green beans. Dark roasted coffee showed the highest values of dry matter, fat, and the major fatty acids among the beans under study. Our results confirmed that coffee beans contain mainly linoleic, palmitic, oleic, and stearic acids. In conclusion, the analysis of the selected chemical parameters allowed for differentiation between green and roasted beans submitted to different degrees of roasting and coffee by-products. The factors obtained by LDA proved that 89.08% of the variability between coffee beans and their by-products correlates with lignoceric, myristic, behenic, tricosanoic, arachidic, and heneicosanoic acids. Light and medium roasting caused a significant decrease in the identified fatty acids. Only behenic, cis-11-eicosenoic, and lignoceric acids showed stability in their content with light and medium roasting temperatures.

Silverskin appeared to be a good source of lignoceric, myristic, and behenic acids and had a higher concentration of dietary fiber than Cascara. Silverskin showed the highest values of the dietary fiber among the studied samples and the second lowest in fat after Cascara. In general, the data confirmed that coffee beans (green and roasted) and their by-products are healthy foods attending to their composition in nutrients, including those studied in the present research. The results suggest that, given the fatty acids content and dietary fiber, coffee by-products might be used for the production of enriched and innovative foods. 

## Figures and Tables

**Table 1:** Detailed description of analyzed samples.

	Samples					
	100% *Coffea arabica*					
	Green	Light Roast	Medium Roast	Dark Roast	Cascara	Silverskin
**Sample ID**	GC	LRC	MRC	DRC	CC	SS
**Origin**	Panama (Hartmann, Santa Clara)				Panama (Hartmann, Santa Clara)	Caffé Oro Ltd. (Zvolen, Slovakia)

Notes: processing (dry, natural), variety (Maragogype), altitude (1300–1500 mamsl), date of harvest of coffee beans and Cascara (2019), date of roast (light, medium, and dark) of coffee beans and Silverskin (December 2021).

**Table 2:** Average content of dry matter, fat, and profile of fatty acids determined by GC-FID.

Sample ID	GC	LRC	MRC	DRC	CC	SS
Dry matter	932.20 ± 0.100 ^a^	959.85 ± 0.050 ^b^	981.20± 0.200 ^c^	983.10 ± 0.100 ^d^	890.50 ± 0.000 ^e^	920.80 ± 0.100 ^f^
Fat	99.42 ± 0.415 ^b^	68.64 ± 0.100 ^d^	73.22 ± 0.115 ^c^	135.84 ± 0.120 ^a^	4.24 ± 0.040 ^f^	23.70 ± 0.290 ^e^
α-linolenic acid	1.38 ± 0.001 ^b^	1.00 ± 0.004 ^d^	1.03 ± 0.000 ^c^	1.97 ± 0.002 ^a^	0.88 ± 0.168 ^e^	0.48 ± 0.006 ^f^
Arachidic acid	2.89 ± 0.005 ^c^	1.85 ± 0.000 ^e^	1.96 ± 0.000 ^d^	3.44 ± 0.010 ^a^	0.14 ± 0.008 ^f^	3.25 ± 0.034 ^b^
Behenic acid	0.71 ^±^ 0.003 ^b^	0.41 ± 0.002 ^c^	0.44 ± 0.000 ^c^	0.69 ± 0.002 ^b^	0.00 ± 0.000 ^d^	4.14 ± 0.038 ^a^
cis-11-eicosenoic acid	0.32 ± 0.000 ^b^	0.24 ± 0.001 ^c^	0.24 ± 0.001 ^c^	0.47 ± 0.002 ^a^	0.00 ± 0.000 ^f^	0.10 ± 0.002 ^e^
Heneicosanoic acid	0.07 ± 0.000 ^a^	0.00 ± 0.000 ^c^	0.00 ± 0.000 ^c^	0.00 ± 0.000 ^c^	0.00 ± 0.000 ^c^	0.07 ± 0.004 ^b^
Heptadecanoic acid	0.10 ± 0.002 ^a^	0.07 ± 0.002 ^c^	0.08 ± 0.000 ^b^	0.00 ± 0.000 ^d^	0.00 ± 0.000 ^d^	0.00 ± 0.000 ^d^
Lignoceric acid	0.26 ± 0.000 ^c^	0.16 ± 0.002 ^d^	0.18 ± 0.001 ^d^	0.29 ± 0.001 ^b^	0.00 ± 0.000 ^e^	0.73 ± 0.005 ^a^
Linoleic acid	43.67 ± 0.009 ^b^	30.19 ± 0.010 ^d^	32.54 ± 0.001 ^c^	60.56 ± 0.041 ^a^	1.32 ± 0.469 ^f^	5.88 ± 0.243 ^e^
Myristic acid	0.07 ± 0.000 ^b^	0.00 ± 0.000 ^c^	0.00 ± 0.000 ^c^	0.00 ± 0.000 ^c^	0.00 ± 0.000 ^c^	0.25 ± 0.018 ^a^
Oleic acid	7.23 ± 0.004 ^b^	5.41 ± 0.025 ^d^	5.83 ± 0.007 ^c^	11.14 ± 0.031 ^a^	0.24 ± 0.069 ^f^	1.12 ± 0.270 ^e^
Palmitic acid	34.93 ± 0.007 ^b^	24.40 ± 0.011 ^d^	25.39 ± 0.005 ^c^	47.61 ± 0.035 ^a^	1.34 ± 0.241 ^f^	6.03 ± 0.059 ^e^
Stearic acid	6.96 ± 0.002 ^b^	4.57 ± 0.001 ^d^	5.05 ± 0.005 ^c^	9.02 ± 0.007 ^a^	0.33 ± 0.122 ^f^	1.46 ± 0.270 ^e^
Tricosanoic acid	0.10 ± 0.001 ^a^	0.00 ± 0.000 ^c^	0.07 ± 0.000 ^b^	0.00 ± 0.000 ^c^	0.00 ± 0.000 ^c^	0.10 ± 0.013 ^a^

Notes: ^a^, ^b^, ^c^, ^d^, ^e^, ^f^ = groups within a row with different superscripts differ significantly at *p* ≤ 0.0001; GC = green coffee, LRC = light roast coffee, MRC = medium roast coffee, DRC = dark roast coffee, CC = Cascara, SS = Silverskin; all parameters are presented in g·kg^−1^, significant in all parameters (<0.0001).

**Table 3:** Average content of fiber and acid and neutral detergent fiber in green and roasted *C. arabica* and its by-products.

Sample ID	Fiber (g·kg^−1^)	ADF (g·kg^−1^)	NDF (g·kg^−1^)
GC	167.29 ± 1.615 ^e^	25.80 ± 1.290 ^e^	47.84 ± 2.055 ^c^
LRC	268.75 ± 0.490 ^c^	37.96 ± 0.190 ^c^	57.64 ± 0.335 ^b^
MRC	284.59 ± 0.310 ^b^	41.82 ± 0.360 ^a^	60.45 ± 1.970 ^a^
DRC	213.45 ± 0.100 ^d^	36.60 ± 1.230 ^d^	41.92 ± 2.305 ^e^
CC	88.34 ± 0.160 ^f^	18.03 ± 1.905 ^f^	16.00 ± 1.585 ^f^
SS	314.95 ± 1.635 ^a^	38.64 ± 0.505 ^b^	44.19 ± 1.450 ^d^
Pr > F(Model)	<0.0001	<0.0001	<0.0001
Significant	**Yes**	**Yes**	**Yes**

Notes: ^a^, ^b^, ^c^, ^d^, ^e^, ^f^ = groups within a column with different superscripts differ significantly at *p* ≤ 0.0001; GC = green coffee, LRC = light roast coffee, MRC = medium roast coffee, DRC = dark roast coffee, CC = Cascara, SS = Silverskin.

## Data Availability

Not applicable.

## Supplementary Materials

The following supporting information can be downloaded at: https://www.mdpi.com/article/10.3390/foods11081082/s1 Detailed description of the fiber determination methodology, Figure S1: Chromatograms of fatty acids measurements by GC-FID.

## Abbreviations

ADF: acid detergent fiber; CC: Cascara coffee; DM: dry matter; DRC: dark roast coffee; EFSA: European Food Safety Authority; FAME: fatty acid methyl esters profile; GC: green coffee; GC-FID: gas chromatography with flame ionization detection; LRC: light roast coffee; Mamsl: meters above mean sea level; MRC: medium roast coffee; MUFA: monounsaturated fatty acids; NDF: neutral detergent fiber; PUFA: polyunsaturated fatty acids; REGWQ: the Ryan, Einot, Gabriel, Welsh, studentized Range Q test; SFA: saturated fatty acids.
