# Supplementary material for: Comparative Analysis of Selected Chemical Parameters of Coffea arabica, from Cascara to Silverskin
Source: Foods. 2022 Apr 8;11(8):1082. doi: 10.3390/foods11081082 (PMC9027595; doi:10.3390/foods11081082)
Supplement: Supplementary file 1 [file foods-11-01082-s001.zip › foods-1637113-supplementary.pdf]

Determination of fiber

Decree of the Ministry of Justice of the Slovak Republic no. 2145/2004-100 of 23.8.2004 on official sampling and laboratory testing and evaluation of feeding stuff [22]. Part H1: Determination of fiber. Part H2: Determination of neutral detergent fiber (NDF). Part H3: Determination of acid-detergent fiber (ADF). The procedure is described in detail in the Supplementary material.

Chemical reagents

1. Determination of fiber: Sulfuric acid H_2SO_4 96%, concentration c=0.13 mol.l^{-1} (Centralchem, Bratislava, Slovakia), preparation of the reagent: 14.6 mL H_2SO_4 96% and 2 L of distilled H_2O
2. Antifoaming reagent n-octanol (Centralchem, Bratislava, Slovakia), Acetone C_3H_6O (Centralchem, Bratislava Slovakia),
3. Sodium hydroxide solution, concentration c=0.23 mol.l^{-1} (Centralchem, Bratislava, Slovakia), preparation of the reagent: 18.5 g NaOH diluted in distilled H_2O (2L

Apparatus

1. Dosi-Fiber Extractor Selecta (Cereal Tester SA, Fleurus, Belgium)
2. Oven (Fisher Scientific, Waltham, USA)
3. Muffle furnace with thermostat (Fisher Scientific, Waltham, USA)

Exactly 1.0000 g (m_0) of the homogenized sample was weighed into a glass crucible and connected to the apparatus. The sample is washed with acetone and several times with hot distilled water. Then the valve under the sample is closed, and hot 0.13 M H_2SO_4 is added. The heating is reduced to 60°C and left to boil slightly for 30 minutes when boiling. The heating is turned off, and the acid is released after 30 minutes, then the sample is washed repeatedly with hot distilled water. Subsequently, the valve is closed, and hot 0.23 M NaOH is added. When it starts boiling, the heating is reduced to 60°C and left to boil gently for 30 min. After that, the hydroxide has been released, and the sample is washed repeatedly with hot distilled water. The crucible with the sample is disconnected from the apparatus, placed in an oven, and dried for 5 hours at 105 °C. Subsequently, the crucible with a sample is cooled in a desiccator, and the crucible with a sample is weighed (m_1). The sample is then placed in a muffle furnace and incinerated at 520 °C for 5 hours after sufficient cooling. The sample is placed in a desiccator. After cooling, weigh, record the weight (m_2).

The fiber is calculated according to the following equation:

$$fiber\ (\%) = \frac{(m_1 - m_2) * 100}{m_0}$$

m_1 is the mass (g) of crucible with sample after drying
m_2 is the mass (g) of crucible with sample after incineration
m_0 is the mass of homogenized sample (1 g)

Determination of neutral detergent fiber (NDF)

Decree of the Ministry of Justice of the Slovak Republic no. 2145/2004-100 of 23.8.2004 on official sampling and laboratory testing and evaluation of feeding stuff [22]. Part H1: Determination of fiber. Part H2: Determination of neutral detergent fiber (NDF). Part H3: Determination of acid-detergent fiber (ADF). The procedure is described in detail in the Supplementary material.

Chemical reagents

Determination of NDF:

1. Sodium sulphite Na_2SO_3 (Centralchem, Bratislava, Slovakia)
2. Sodium hydroxide NaOH (Centralchem, Bratislava, Slovakia),
3. Chelaton III, disodium salt of ethylenediaminetetraacetic acid $Na_2EDTA.2H_2O$ (Centralchem, Bratislava, Slovakia),
4. Sodium tetraborate, decahydrate, $Na_2B_4O_7$. 10 H_2O (Centralchem, Bratislava, Slovakia),
5. Disodium hydrogen phosphate, Na_2HPO_4 (Centralchem, Bratislava, Slovakia),
6. Sodium lauryl sulfate (Sigma-Aldrich, Steinheim Germany),

7. Ethylene glycole (Centralchem, Bratislava, Slovakia).

The neutral detergent reagent is prepared as follows: Separately, to 4 volumetric flasks, precisely 8 g of NaOH, 29.2 g of Chelaton III, 13.6 g Sodium tetraborate, decahydrate, $Na_2B_4O_7$. 10 H_2O, and 9.12 g of Disodium hydrogen phosphate, Na_2HPO_4 are weighted. Subsequently, 36 g of Sodium lauryl sulfate is weighted to 1 L volumetric flask, and 600 mL of distilled H2O is added, and the solution is boiled. Weighted chemical from the first step: Chelaton III, Sodium tetraborate, decahydrate, $Na_2B_4O_7$. 10 H_2O and Disodium hydrogen phosphate Na_2HPO_4 are diluted with hot distilled water and poured into a 2 L volumetric flask. The sodium lauryl sulfate solution needs to be kept warm; when cold, it solidifies. On the following day, 40 mL of ethylene glycol is added to the solution for conservation purposes. Then the volumetric flask is filled up with distilled water. Solution pH must be 7,00. If necessary, pH can be adjusted by 72% H_2SO_4 (approx. 5 mL).

Apparatus
1. Dosi-Fiber Extractor Selecta (Cereal Tester SA, Fleurus, Belgium)
2. Oven (Fisher Scientific, Waltham, USA)
3. Muffle furnace with thermostat (Fisher Scientific, Waltham, USA)

Exactly 1.0000 g of the homogenized sample into a glass crucible, and a small amount of Na_2SO_3 is added to the dry sample (tip of a teaspoon). The crucible with the sample is connected to the apparatus. Then the valve under the sample is closed, a cold solution of neutral detergent is added, the heating is switched on, and when it starts to boil, the heating is reduced to 60 °C and is left to boil slightly for 1 hour. The heating is turned off, the NDV reagent is drained, the sample is washed repeatedly with hot distilled water, and the valve is closed. The crucible with a sample is disconnected, placed in an oven, and dried for 5 hours at 105 °C. Then the crucible with a sample is allowed to cool in a desiccator and weighted (m_1). The sample is then placed in a muffle furnace and incinerated at 520 °C for 5 hours after sufficient cooling. A sample is placed in a desiccator. After cooling, weigh, record the weight in m_2.

The fiber is calculated according to the following equation:

$$NDF\ (\%) = \frac{(m_1 - m_2) * 100}{m_0}$$

m_1 is the mass (g) of crucible with sample after drying
m_2 is the mass (g) of crucible with sample after incineration
m_0 is the mass of homogenized sample (1 g)

Determination of acid detergent fiber (ADF)

Decree of the Ministry of Justice of the Slovak Republic no. 2145/2004-100 of 23.8.2004 on official sampling and laboratory testing and evaluation of feeding stuff [22]. Part H1: Determination of fiber. Part H2: Determination of neutral detergent fiber (NDF). Part H3: Determination of acid-detergent fiber (ADF). The procedure is described in detail in the Supplementary material.

Chemical reagents
1. Sulfuric acid H_2SO_4 96% (Centralchem, Bratislava, Slovakia),
2. Cetyltrimethylammonium bromide CETAB (Sigma-Aldrich, Steinheim Germany),
3. Distilled water.

ADF reagent is prepared as follows:
56.5 mL of 96% H_2SO_4 is diluted in 500mL of distilled H_2O (in 2 L volumetric flask, which is in water bath due to cooling). 40g of CETAB is mixed with 50 mL of distilled water (in 1L volumetric flask). The content of the volumetric flask is stirred till the foam appears, then 500 mL of distilled water is added, and the content is heated till is start boiling. Cooled solution of CETAB is poured into the prepared H_2SO_4 solution and filled up with distilled water.

Exactly 1.0000 g of the homogenized sample into a glass crucible. The crucible with the sample is connected to the apparatus. Then the valve under the sample is closed, a cold solution of acid-detergent reagent is added, the heating is

switched on, and when it starts to boil, the heating is reduced to 60 °C and is left to boil slightly for 1 hour. The heating is turned off, the ADV reagent is drained, the sample is washed repeatedly with hot distilled water, and the valve is closed. The crucible with a sample is disconnected, placed in an oven, and dried for 5 hours at 105 °C. Then the crucible with a sample is allowed to cool in a desiccator and weighted (m_1). The crucible with a sample is then placed in a muffle furnace and incinerated at 520 °C for 5 hours after sufficient cooling. The sample is placed in a desiccator. After cooling, weigh, record the weight (m_2).

Apparatus

1. Dosi-Fiber Extractor Selecta (Cereal Tester SA, Fleurus, Belgium)
2. Oven (Fisher Scientific, Waltham, USA)
3. Muffle furnace with thermostat (Fisher Scientific, Waltham, USA)

The fiber is calculated according to the following equation:

$$ADF\ (\%) = \frac{(m_1 - m_2) * 100}{m_0}$$

m_1 is the mass (g) of crucible with sample after drying
m_2 is the mass (g) of crucible with sample after incineration
m_0 is the mass of homogenized sample (1 g)

Figure S1 Chromatograms of fatty acids measurements by GC-FID

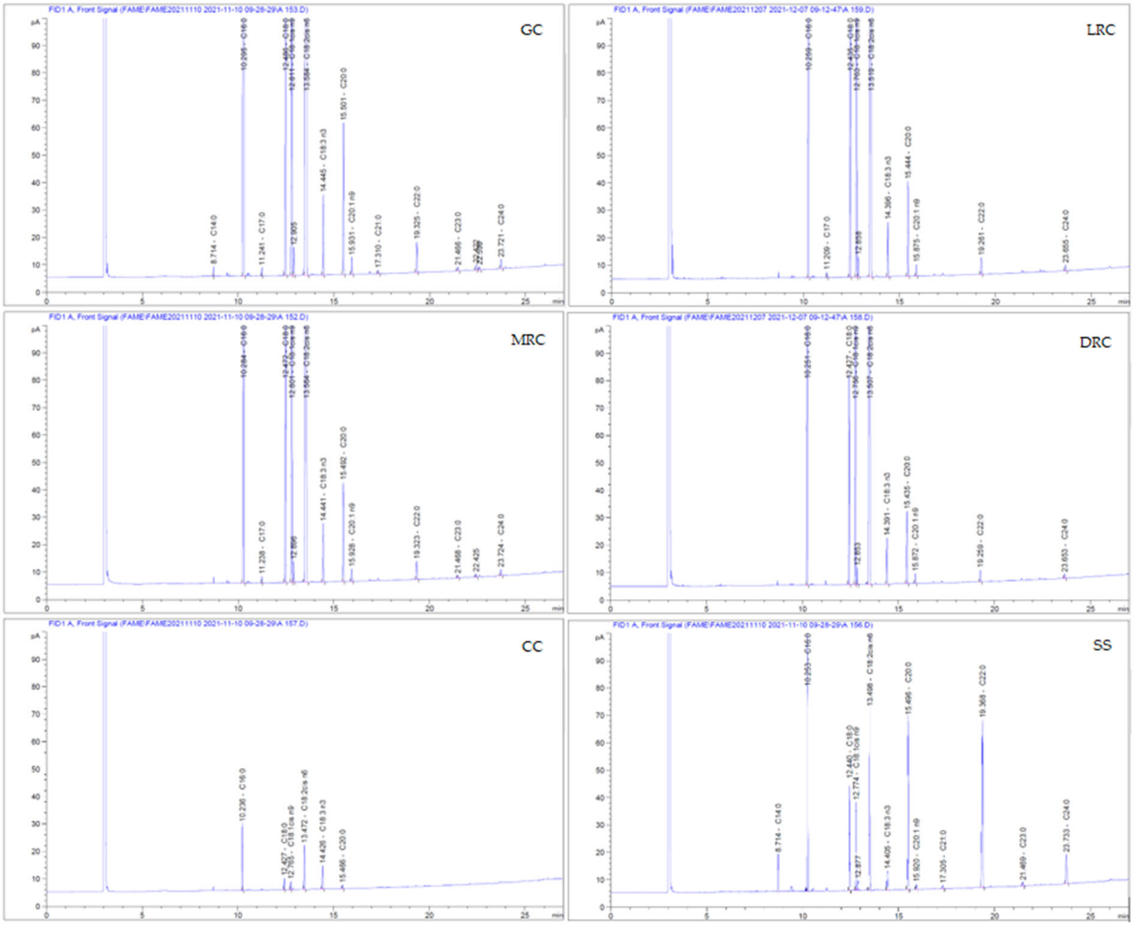

Note: GC- green coffee, LRC – light roasted coffee, MRC- medium roasted coffee, DRC – dark roasted coffee, CC – Cascara, SS - Silverskin
